# Cancer Predisposition Genes in Adolescents and Young Adults (AYAs): a Review Paper from the Italian AYA Working Group

**DOI:** 10.1007/s11912-022-01213-3

**Published:** 2022-03-23

**Authors:** Angela Toss, Paola Quarello, Maurizio Mascarin, Giuseppe Luigi Banna, Marco Zecca, Saverio Cinieri, Fedro Alessandro Peccatori, Andrea Ferrari

**Affiliations:** 1 Department of Oncology and Hematology, Azienda Ospedaliero-Universitaria di Modena, Modena, Italy; 2 Department of Medical and Surgical Sciences for Children and Adults, University of Modena and Reggio Emilia, Modena, Italy; 3 Paediatric Onco-Haematology, Stem Cell Transplantation and Cellular Therapy Division, Regina Margherita Children’s Hospital, Turin, Italy; 4 Department of Public Health and Paediatric Sciences, University of Torino, Turin, Italy; 5 AYA Oncology and Pediatric Radiotherapy Unit, Centro di Riferimento Oncologico IRCCS, Aviano, Italy; 6 Candiolo Cancer Institute, FPO-IRCCS, SP142, km 3.95, 10060, Candiolo, Turin, Italy; 7 Department of Pediatric Hematology/Oncology, Fondazione IRCCS Policlinico San Matteo, Pavia, Italy; 8 Medical Oncology Unit and Breast Unit Ospedale Perrino ASL, Brindisi, Italy; 9 Fertility and Procreation Unit, Gynecologic Oncology Program, European Institute of Oncology IRCCS, Milan, Italy; 10 Pediatric Oncology Unit, Fondazione IRCCS Istituto Nazionale dei Tumori, Via Venezian 1, 20133 Milan, Italy

**Keywords:** Adolescents, Young adults, Cancer, Predisposition genes

## Abstract

**Purpose of Review:**

The present narrative systematic review summarizes current knowledge on germline gene mutations predisposing to solid tumors in adolescents and young adults (AYAs).

**Recent Findings:**

AYAs with cancer represent a particular group of patients with specific challenging characteristics and yet unmet needs. A significant percentage of AYA patients carry pathogenic or likely pathogenic variants (PV/LPVs) in cancer predisposition genes. Nevertheless, knowledge on spectrum, frequency, and clinical implications of germline variants in AYAs with solid tumors is limited.

**Summary:**

The identification of PV/LPV in AYA is especially critical given the need for appropriate communicative strategies, risk of second primary cancers, need for personalized long-term surveillance, potential reproductive implications, and cascade testing of at-risk family members. Moreover, these gene alterations may potentially provide novel biomarkers and therapeutic targets that are lacking in AYA patients. Among young adults with early-onset phenotypes of malignancies typically presenting at later ages, the increased prevalence of germline PV/LPVs supports a role for genetic counseling and testing irrespective of tumor type.

## Introduction

Adolescents and young adults (AYAs) with cancer represent a particular group of patients whose specific challenging characteristics are currently recognized by the scientific community. According to the most recent definition [1, 2], the AYA population is defined as those subjects diagnosed with cancer at ages 15 through 39. This represents a heterogeneous and challenging group of patients with increasing cancer incidence, modest survival gains compared with other age groups, and unique and often unmet needs [3, 4]. Tumor in AYAs shows substantial differences in etiology, cancer type, molecular profile, psychosocial implications, prognosis, and long-term treatment side effects from cancer affecting other age groups [4–7], and relevant differences persist also across AYA age groups themselves [8].

Various AYA oncology programs have been developed in the last year in several parts of the world (involving numerous organizations, healthcare providers, academic societies, and governments) [7, 9]. In particular, the European adult and pediatric oncology societies—the European Society for Medical Oncology (ESMO) and the European Society for Paediatric Oncology (SIOPE)—established a joint Working Group dedicated to AYA, with the aims of increasing awareness among the scientific community, exchanging knowledge, and foreseeing integrated programs to improve the standard of care for AYA with cancer across Europe [4•]. In the wake of this experience, also in Italy, a collaboration between pediatric and adult oncologists on the AYA theme has been recently formalized, and in April 2021, the national adult medical oncology society (AIOM—Associazione Italiana di Oncologia Medica) joined the pediatric hematology-oncology group (AIEOP—Associazione Italiana Ematologia Oncologia Pediatrica) in the creation of a formal AIEOP-AIOM Working Group dedicated to AYAs. Among the different initiatives of this group, it has been decided to focus on the specific need of counseling and genetic testing, as essential part of cancer journey of AYA patients and their family. To implement this aspect in Italian centers, we present a review of the cancer predisposition genes in this age range. Since the new-born AIEOP-AIOM Working Group involves pediatricians specialized in hematology-oncology and medical oncologists, who in Italy treat solid neoplasms, the work focuses on solid cancers rather than hematologic disorders. This topic appears relevant because it is known that a significant percentage of AYA patients carry pathogenic or likely pathogenic variants in cancer predisposition genes. A recent analysis of 1,507 patients with solid tumors showed 12% of germline pathogenic and/or likely pathogenic variants (PV/LPVs) in known cancer-predisposing genes [10••]. Nevertheless, this study also included children and only AYAs under 29 years of age. Previous studies showed that 7–8% of patients diagnosed <20 years of age have PV/LPV in known cancer predisposition genes, with adrenocortical carcinoma (50%) and high-grade glioma (25%) having the highest percentage of variants [11–14].

The presence of a germline PV/LPV in known cancer-predisposing genes brings several implications of utmost importance. First, germline variants may provide novel biomarkers and therapeutic targets that are lacking in AYA patients, as recently happened with the introduction of PARP inhibitors [15•]. Second, mutation carriers present elevated risk of secondary neoplasms that need specific surveillance programs [16]. Third, the identification of a hereditary cancer syndrome has an impact on all the relatives carrying the same mutation in terms of primary and secondary prevention. Despite all these implications, knowledge on spectrum, frequency, and implications of germline variants in AYAs with solid tumors is limited. Therefore, the objective of this systematic review was to summarize current knowledge regarding genes predisposing to solid tumors in AYA patients.

## Methods

**Fig. 1:**
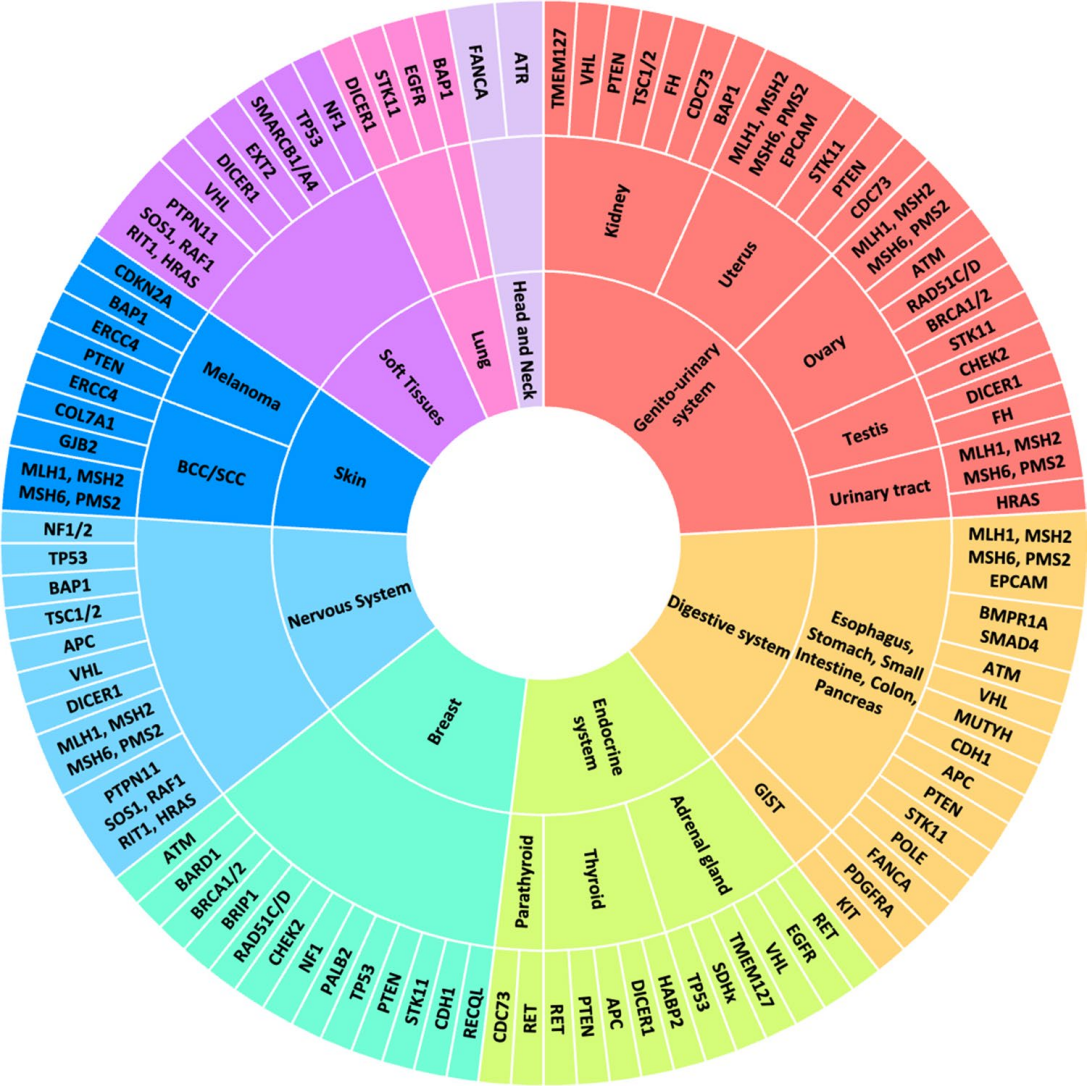
From the tumor to the gene. The figure represents the principal predisposition genes associated with increased risk of cancer in each organ and apparatus

The search was carried out in the PubMed database (http://www.ncbi.nlm.nih.gov/pubmed (accessed date 6 April 2021)). Key search terms used were as follows: “germline” AND “cancer” OR “neoplasm” AND “adolescent” OR “young adult” OR “AYA.” The eligibility criteria for articles reviewed included all types of articles published from January 2018 on AYA patients with a diagnosis of solid neoplasm that was associated with a germline PV/PLV in any cancer susceptibility gene. Then, reference lists were examined. Studies published in a language other than English were excluded. The systematic search identified the main predisposition genes described in the present review and summarized in Fig. 1.

## Cancer Predisposition Genes in AYA

### Genes Involved in the DNA Double-Strand Break Repair Mechanism

#### *ATM* (Ataxia Telangiectasia Mutated)

Homozygous or compound heterozygous *ATM* mutations cause ataxia telangiectasia, a syndrome characterized by progressive cerebellar ataxia, oculomotor apraxia, immunodeficiency, and general increased risk of malignancies [17] with an overall cumulative incidence of cancer by age 40 of 38.2% [18]. On the other hand, heterozygous *ATM* germline PV/LPV can be found in 0.35–1% of the general population [19] and are associated with an increased risk for breast cancer (BC) at a higher median age of onset (46.9 years) [20]. Maxwell et al. [21] identified 8 patients with a *ATM* germline PV/LPV out of 278 (2.9%) *BRCA1/2*-negative patients with BC diagnosed at less than 40 years of age. Moreover, a significant association between *ATM* heterozygous PV/LPV and ovarian cancer [22, 23], pancreatic cancer [24], and prostate cancer [25] has been described.

#### *ATR* (Ataxia Telangiectasia and Rad3 Related)

Mutations in *ATR* gene are rare. Homozygous hypomorphic mutations in *ATR* have been detected in Seckel syndrome, characterized by developmental delay and premature aging [26]. A different kind of genetic disorder due to heterozygous mutations in *ATR* comprises skin telangiectasis; mild developmental anomalies of the hair, teeth, and nails; and an increased risk of oropharyngeal cancer typically in the third decade of life or thereafter [27]. Other malignancies reported included nonmelanoma skin cancer, breast cancer, and cervical cancer, although data on these associations are still conflicting [28, 29].

#### *BARD1* (BRCA1 Associated RING Domain 1)

*BARD1* is a BC moderate-risk gene [30, 31] with a lifetime risk approximately doubled than that in the general population. Deleterious *BARD1* germline variants are significantly associated with early-onset BC. The mean age at first BC diagnosis in *BARD1* mutation carriers was 42.3 years (range 24–60 years) in a German cohort [32]. Particularly, two recent studies confirmed that *BARD1* PV/LPVs are enriched among triple-negative BC patients compared to other BC subtypes [33, 34].

#### *BRCA1* (BReast CAncer Gene 1) and BRCA2 (Breast Cancer Gene 2)

Germline mutations in the tumor suppressor genes *BRCA1* and *BRCA2* account for most cases of hereditary breast and ovarian cancer syndrome [35]. The population frequency of *BRCA1*/*2* PV/LPV has been historically estimated 1:400, except for populations with high-frequency founder mutations, such as the Ashkenazi Jewish population [36]. However, recent unselected population-based genomic screening efforts have found a higher, almost doubled prevalence (1:190), predominantly in European ancestry individuals [37]. The cumulative BC risk to age 40 years in women was estimated 24% (95%CI, 21–29%) for *BRCA1* and 13% (95%CI, 9–19%) for *BRCA2* carriers [38], whereas the cumulative BC risk to age 40 in men was 0.12% (95%CI, 0.012–0.58%) for *BRCA1* and 1.2% (95%CI, 0.3–3.6%) for *BRCA2* carriers [39]. OC risk is almost null until 30 years and remains low from 31 to 40 years: 2% (95%CI, 1–3%) for *BRCA1* and 0% (95%CI, 0–2%) for *BRCA2* carriers [38]. In AYA with PV/LPV in *BRCA1/2*, the risk for prostate and pancreatic cancer is negligible [40, 41]. On these bases, surveillance in female *BRCA* carriers should start at 25 years of age with breast MRI, and at the same time, preventive surgeries can be discussed [42].

#### *BRIP1* (BRCA1 Interacting Protein C-terminal Helicase 1)

Pathogenic mutations in *BRIP1* have been described for the first time in two patients with early-onset BC [43]. Weber-Lassalle et al. [44] observed a barely significant association of *BRIP1* PV/LPV mutations with BC in the subgroup of patients with an age at first diagnosis < 61 years. However, the role of pathogenic *BRIP1* mutations in BC risk remains conflicting [31, 45]. OC risk is almost null until 40 years [46].

#### *RAD51C* and *RAD51D* (RAD51 Recombinase Paralog C and Paralog D)

Genetic testing through multigene cancer panel revealed an association between PV/LPV in *RAD51C* and *RAD51D* and the increased risk of OC [47, 48]. The estimated cumulative risk of developing OC to age 40 years was 0.2% (95%CI, 0.08–0.4%) for a woman with a *RAD51C* PV/LPV and 0.1% (95%CI, 0.06–0.3%) for a woman with a *RAD51D* PV/LPV. The relative risk of OC in the third decade was 2.85 (95%CI, 0.46–17.70) in carriers of *RAD51C* PV/LPV and 3.60 (0.78 to 16.75) in carriers of *RAD51D* PV/LPV [49]. Recent findings highlighted the evidence of an association between protein-truncating variants of *RAD51C/D* and BC risk [31, 50]. The estimated cumulative risk of developing BC to age 40 years was 1% (95%CI, 0.7–1%) for a woman with a *RAD51C* PV/LPV and 0.9% (95%CI, 0.6–1%) for a woman with a *RAD51D* PV/LPV. The relative risk of BC in the third decade was 3.25 (95%CI, 1.60–6.62) in carriers of *RAD51C* PV/LPV; in carriers of RAD51D PV/LPV, the relative risk of BC between 20 and 39 years of age was 2.25 (95%CI, 1.25–4.04) [49].

#### *CHEK2* (Checkpoint Kinase 2)

*CHEK2* has the highest mutation prevalence in individuals of European descent, while the spectrum and frequency of pathogenic variants vary among specific European populations [51]. Different case-control studies had revealed a significant association between *CHEK2* 1100delC mutation and early-onset BC [52]. In a Swedish cohort, the mean age at diagnosis of *CHEK2* 1100delC carriers was 12 years lower than that of non-carriers (46 vs 58 years, *p*=0.001) [52] and this has been recently confirmed in an Italian cohort of *CHEK2* mutation carriers (median age at first BC onset 46.1 years) [20]. Relative risk of developing BC to age 35 years was 2.59 (95%CI, 1.23–5.47) for *CHEK2* 1100delC carriers, whereas the cumulative risk to age 40 years was < 5% [51]. Greville-Heygate et al. [53] detected 53 (2.3%) patients carrying a germline *CHEK2* PV/LPV out of 2344 women with early-onset BC and *CHEK2*-associated tumors showed a worse prognosis. Moreover, a case-control enrichment analysis recently provided evidence for *CHEK2* as a novel moderate-penetrance testicular germ cell tumor susceptibility gene [54]. Finally, pathogenic *CHEK2* variants were associated with an increased risk of other malignancies including colon, prostate, kidney, bladder, and thyroid cancers, according to specific mutations (frameshift or missense substitutions) at a more mature age [55].

#### *PALB2* (Partner and Localizer of BRCA2)

*PALB2* is a BC susceptibility gene [56]. The risk of BC for women with a PALB2 PV/LPV was 8 to 9 times as high among those younger than 40 years of age compared with the general population with a cumulative risk estimated to be 14% (95%CI, 9–20) by 50 years of age [57]. Some studies highlight a possible association between PALB2 mutations and OC and pancreatic cancer [58].

#### *FANCA* (Fanconi Anemia Complementation Group A) Family

Fanconi anemia (FA) is a rare autosomal recessive genetic disorder that comprises a broad spectrum of clinical features of variable penetrance, mainly progressive bone marrow failure, congenital abnormalities, and cancer predisposition [59]. Until now, 22 genes have been described as FA genes: *FANCA*, *FANCB*, *FANCC*, *FANCD1/BRCA2*, *FANCD2*, *FANCE*, *FANCF*, *FANCG/XRCC9*, *FANCI*, *FANCJ/BRIP1*, *FANCL/PHF9*, *FANCM*, *FANCN/PALB2*, *FANCO/RAD51C*, *FANCP/SLX4*, *FANCQ/ERCC4*, *FANCR/RAD51*, *FANCS/BRCA1*, *FANCT/UBE2T*, *FANCU/XRCC2*, *FANCV/REV7*, and *FANCW/RFWD3* [60]. FA patients develop acute myeloid leukemia at an incidence 700-fold higher compared to the general population. The median age at diagnosis is 19 (range 16–27) years, with a cumulative incidence of 10% by 50 years of age. FA patients who overcome severe bone marrow failure following a successful bone marrow transplant are still likely to develop solid tumors (head and neck, esophageal, gastrointestinal, vulvar, and anal cancers) at an incidence approximately 50-fold higher, with a median onset age of 30 (range 4–44) years and a cumulative risk of 10% by 40 years of age [61, 62]. Finally, heterozygous mutations in FA genes (e.g., *BRCA1*, *BRCA2*, *BRIP1*, *PALB2*, and *RAD51C*) are associated with hereditary breast and/or ovarian cancer predisposition (paragraphs above) [63].

### Genes Involved in the Nucleotide Excision Repair (NER) Mechanism

#### *ERCC4* (Human Excision Repair Cross-complementing Rodent Repair Deficiency, Complementation Group 4)

*ERCC4* mutations are associated with three clinically distinct disorders: xeroderma pigmentosum, XFE progeroid syndrome, and Fanconi anemia [64, 65]. In xeroderma pigmentosum, there is a 1000-fold increased frequency of early-onset basal cell or squamous cell carcinomas and melanomas of the skin, often with multiple primary tumors, by age 20. The median age at first skin neoplasm diagnosis is 8 years, nearly 50 years younger than that found in the general population. A 5% risk of malignant melanoma is reported [66].

#### *POLE* (DNA Polymerase Epsilon)

Germline missense pathogenic variants in the exonuclease domain of polymerases epsilon (*POLE*) predispose to multiple colorectal adenomas and carcinomas, causing the so-called polymerase proofreading–associated polyposis (MIM 615083; 612591) [67]. Evidence of extracolonic tumors has been reported, including endometrial, brain, breast, ovarian, stomach, pancreas, and skin tumors, among others [68–70].

### Genes Involved in the Mismatch Mediated Repair (MMR) Mechanism

#### *MLH1* (MutL Homolog 1), *MSH2* (MutL Homolog 2), *MSH6* (MutS Homolog 6), *PMS2* (PMS1 Homolog 2)

Germline heterozygous PV/LPVs in *MLH1*, *MSH2*, *MSH6*, or *PMS2* cause Lynch syndrome (LS). LS, also called hereditary nonpolyposis colorectal cancer (HNPCC), leads to various types of tumors, including most of all colorectal (CRC) and endometrial cancers, but also ovarian, stomach, small bowel, urinary tract, biliary tract, brain, skin (sebaceous adenomas, sebaceous carcinomas, and keratoacanthomas), pancreatic, and prostate cancers [71]. The *MLH1* variant is correlated with the highest risk of developing CRC with a cumulative incidence at 40 years of age of 15.3% in females and 18.9% in males, and a cumulative incidence of endometrial cancer of 1.9% at 40 years [72]. PV/LPVs in *MSH2* are correlated with the second highest risk of CRC and the highest risk of developing other non-colorectal cancers, with cumulative cancer incidences at 40 years of 6.9% for CRC in females, 9.9% for CRC in males, and 2.3% for endometrial cancer [72]. Cancers in *MSH6* mutation carriers occur later than those in the *MLH1* or *MSH2* PV/LPVs, with cumulative CRC incidences at 40 of 2.5% (females) and 6.3% (males), and 2.3% for endometrial cancer. The cumulative incidence of overall cancers for *PMS2* mutation carriers is the lowest among the four genes, with cumulative cancer incidences of 0% at the age of 40. On these bases, the Lynch syndrome surveillance should start at 20–25 years of age with colonoscopy and, in selected individuals, upper endoscopy, urinalysis, and physical and neurologic examination may be considered starting between 25 and 40 years of age. Hysterectomy and bilateral salpingo-oophorectomy should be individualized [73, 74].

#### RECQL (RecQ Like Helicase)

*RECQL* was first identified as a novel breast cancer susceptibility gene in 2015, by two independent research groups [75, 76]. In a cohort of early-onset BC patients from Poland (<40 years), the increased risk of BC in carriers of the *RECQL* mutation was found to be 1.9 (95%CI 0.27–13.6) [77]. Nevertheless, several subsequent studies have failed to support the association [78]. No high-quality penetrance study showed statistical significance for additional diseases beyond BC.

### Genes Involved in the MAP Kinase Pathway

#### *PTPN11*, *SOS1, RAF1, RIT1*, *KRAS*, *NRAS*, *BRAF*, *MAP2K1*, *RRAS*, *RASA2*, *A2ML1*, *SOS2*, *LZTR1*

The RASopathies are a collective group of phenotypically related conditions caused by germline PV/LPV in genes within the Ras/mitogen-activated protein kinase (Ras/MAPK) signaling pathway. RASopathy conditions, such as Noonan syndrome (NS; MIM# 163950), cardiofaciocutaneous syndrome (CFC; MIM# 115150), and Costello syndrome (CS; MIM# 218040), typically present with multiple phenotypic features, including poor growth, cardiac anomalies, ectodermal abnormalities, neurodevelopmental deficits, and increased tumor risk [79, 80]. NS is caused by germline mutations of *PTPN11* (50%); *SOS1* (13%); *RAF1* (5%); *RIT1* (5%); or more rarely, *KRAS*, *NRAS*, *BRAF*, *MAP2K1*, *RRAS*, *RASA2*, *A2ML1*, *SOS2*, or *LZTR1* [81]. Children with NS are at an approximately 8-fold increased risk for a spectrum of different cancers [82]. These include (but are not limited to) gliomas such as dysembryoplastic neuroepithelial tumors, acute lymphoblastic leukemia, neuroblastoma, and rhabdomyosarcoma [80, 82–84].

CFC syndrome is due to germline mutation of *KRAS*, *MAP2K1*, *MAP2K2*, or *BRAF* [81]. Affected persons have NS features and tend to have significant mental and neurologic impairment, more severe ectodermal involvement, and characteristic facies. Several cases of childhood cancer have been reported, and the cancer risk may be mildly increased [80, 82].

The CS is due to germline mutations of *HRAS* [85]. In addition to NS features, CS patients have mental deficits, poor feeding, hypertrophic cardiomyopathy, tachycardia, typical skin and hair, a coarse face, and a high childhood cancer risk, especially for embryonal rhabdomyosarcoma (ERMS), NBL, and early-onset bladder cancer. The occurrence of bladder carcinoma in adolescents is distinctly unusual as this is typically a neoplasm of older adults and is not seen with increased frequency in other tumor predisposition syndromes. The cumulative incidence of cancer is 15% by age 20 years [80, 82, 86, 87]. The *HRAS* G12A mutation appears to be associated with the highest cancer risk [88].

#### *NF1* (Neurofibromatosis type 1) and *NF2* (Neurofibromatosis type 2)

Neurofibromatosis (NF1) is a dominantly inherited syndrome with variable disease manifestations, affecting multiple organs, childhood development, and neurocognitive status. NF1 causes significantly increased malignancy risks compared with the general population. Specifically, mutation in NF1 gene is associated with highly elevated risk of malignant peripheral nerve sheath tumors (MPNST), rhabdomyosarcoma, and primary brain tumors. Because of the risk of MPNST being associated with high internal tumor burden, whole-body MRI should be considered between ages 16 and 20 years [89]. Furthermore, women with NF1 ages 30 to 50 showed an increased breast cancer risks of 4- to 5-fold [90].

Neurofibromatosis type 2 (NF2, also known as central neurofibromatosis) is an autosomal dominant disorder that is distinct from NF1 on both genetic and clinical grounds. Around 30% of NF2 presents symptomatically in childhood and nearly 50% by 20 years of age. A hallmark of NF2 is the occurrence of bilateral schwannomas that affect the vestibular branch of the eighth cranial nerve (acoustic neuromas). NF2 patients are also at elevated risk for meningiomas, spinal schwannomas, and ependymomas [89].

### Genes Involved in the Cell Cycle Regulation

#### *CDKN2A* (Cyclin-Dependent Kinase Inhibitor 2A)

*CDKN2A* germline mutations are associated with a 65-fold increase in the risk of melanoma development [91], and these have been identified in approximately 20–40% of families showing a predisposition to melanoma [92, 93]. Mean age at melanoma diagnosis is earlier in *CDKN2A* mutation carriers than in the general population (mean of 35 years vs 59 years). Lifetime penetrance of *CDKN2A* mutations is 0.58 in Europe, 0.76 in the USA, and 0.91 in Australia [94]. Accordingly, the median age of melanoma diagnosis was also younger in Australian melanoma-prone families compared to European families [95]. Importantly, *CDKN2A* mutation carriers have been reported to be at increased risk of developing other early-onset cancers, including breast, lung, pancreatic, and nonmelanoma skin cancers and soft tissue sarcomas [96]. These additional cancer risks are not consistently observed, and this may indicate that the risk of other cancers varies with the specific PV/LPV [97, 98].

#### *TP53* (Tumor Protein 53)

Germline PV/LPVs of *TP53* gene have a high penetrance and cause Li-Fraumeni Syndrome (LFS). LFS is associated with increased risk of breast cancer, soft tissue sarcoma, osteosarcoma, leukemia, brain tumors, adrenocortical carcinoma, and other cancers [99, 100]. The cancer risk imparted by *TP53* mutations is evident at an early age, with female carriers having a cumulative 49% risk of developing cancer by the age of 30, and men having a 21% cancer risk at the same age [101]. Indeed, regardless of familial history, the rate of disease associated with germline *TP53* PV/LPV has been estimated to be between 3.8 and 7.7% in females with breast carcinoma before 31 years of age [102]. On these bases, the Li-Fraumeni syndrome surveillance should start from the birth with US of abdomen and pelvis, neurologic examination, and possibly whole-body and brain MRI, at 18 years of age with dermatologic examination, at 20 with breast MRI, and at 25 with colonoscopy and upper endoscopy [74].

#### *BAP1* (BRCA1-Associated Protein 1)

Heterozygous germline mutations of *BAP1* confer increased susceptibility for the development of several tumors, mostly uveal and cutaneous melanomas, epithelioid atypical Spitz tumors, and mesotheliomas but also other neoplasms, including renal cell carcinoma, lung adenocarcinoma, and meningioma (BAP1-TPDS, OMIM 614327) [103]. However, the complete tumor spectrum associated with germline *BAP1* mutations is still uncertain.

The prevalence of germline *BAP1* alterations in unselected patients with metastatic uveal melanoma ranges from 2 to 8% [104], whereas the prevalence in patients with mesothelioma was 4.4% [105]. BAP1 mutation carriers showed a lower age at diagnosis in comparison with the general population, and median age of onset associated with null variants was younger than that with missense variants (in null variants: 53 years for uveal melanoma, 55 years for mesothelioma, 39 years for cutaneous melanoma, 50 years for renal tumors, and 44 years for nonmelanoma skin cancer) [106].

#### *CDC73* (Cell Division Cycle 73)

Because of its incomplete penetrance, patients with germline *CDC73* mutation can present with a spectrum of phenotypes including seemingly sporadic parathyroid cancer (*CDC73* PV/LPVs have been identified in 20–29% of parathyroid carcinomas), familial isolated hyperparathyroidism (FIHP) with or without parathyroid cancer, or full expression of hyperparathyroidism-jaw tumor syndrome (HPT-JT) [107, 108]. HPT-JT is a rare autosomal dominant syndrome with typical onset in late adolescence or early adulthood that causes familial hyperparathyroidism associated with ossifying fibromas of the maxillofacial bones and increased risk of parathyroid carcinoma. Renal abnormalities occur in 15% of patients and include Wilms’ tumors, hamartomas, renal cell carcinoma, and polycystic disease [109]. Uterine tumors affect up to 75% of female HPT-JT patients and may be benign or malignant (e.g., adenosarcomas) [110].

### Genes Encoding for Transmembrane Receptors

#### *EGFR* (Epidermal Growth Factor Receptor)

*EGFR* germline mutations, including the mutations p.T790M and p.R776H in exon 20 and p.V843I in exon 21, are associated with genetic susceptibility to lung cancer [111–114]. Overall, the estimated risk of developing lung cancer among nonsmoking *EGFR* T790M carriers is 31%, compared with a 0.2% risk in a general population of nonsmokers and an approximately 23% risk in a general population of smokers [115]. Diagnosis of lung adenocarcinoma was accelerated 9.0 years (95%CI, 0.5–16.5 years) by EGFR germline PV/LPV [116, 117].

Furthermore, *EGFR* could be a novel underlying germline predisposition factor for adrenocortical carcinoma (ACC) especially in the AYA population [117].

#### *EPCAM* (Epithelial Cell Adhesion Molecule)

Deletions of *EPCAM* can cause Lynch syndrome through epigenetic silencing of *MSH2* in EPCAM-expressing tissues, resulting in tissue-specific MSH2 deficiency. Carriers of an *EPCAM* deletion had a 75% cumulative risk of colorectal cancer before the age of 70 years (mean age at diagnosis 43 years). Women with *EPCAM* deletions had a 12% cumulative risk of endometrial cancer (mean age at diagnosis 47 years) [118].

#### *KIT* (Receptor Tyrosine Kinase)

Somatic mutations of *KIT* are frequently found in mastocytosis and gastrointestinal stromal tumor (GIST), while germline mutations of *KIT* are rare, and only found in few cases of familial GIST and mastocytosis [119]. GISTs are reported predominantly in patients who are 40 to 70 years old but in rare cases may occur in younger persons. Beghini et al. studied an Italian family in which 4 members over 3 generations, including a father and son, had multiple hyperpigmented spots. At 18 years of age, the father developed multiple GISTs with diffuse hyperplasia of the myenteric plexus. The proband was the 14-year-old son whose hyperpigmented lesions were found to be cutaneous mastocytosis [120].

#### *RET* (REarranged During Transfection)

Inherited mutations in the *RET* proto-oncogene, which encodes a receptor tyrosine kinase, predispose individuals to the multiple endocrine neoplasia type 2 (MEN 2) cancer syndromes. The major component tumor of these syndromes is medullary thyroid carcinoma (MTC) [121]. Different mutations in the *RET* gene produce varying phenotypes for the disease, including age of onset and aggressiveness of MTC, and the presence or absence of other endocrine neoplasms, such as pheochromocytoma or hyperparathyroidism.

*RET* mutations can be classified into 3 groups based on aggressiveness of MTC or level of risk. Level 1 *RET* mutations (codons 609, 768s790, 791, 804, and 891) are the lowest risk for aggressive MTC marked by later onset of tumor development and a more indolent biological course. Patients with level 1 mutations rarely develop tumors before the age of 10 years of age. Level 2 *RET* mutations (codons 611, 618, 620, and 634 mutations) are considered high risk for aggressive MTC. Patients with level 2 *RET* mutations should undergo thyroidectomy before age 5 years. Level 3 *RET* mutations (codons 883, 918, and 922) are the most aggressive of all the *RET* mutations. Patients with level 3 mutations can have metastasis in the first years of life [122].

#### *PDGFRA* (Platelet-Derived Growth Factor Receptor Alpha)

Familial gastrointestinal stromal tumor (GIST) is a rare autosomal dominant genetic disorder associated with *KIT* and *PDGFRA* germline mutations. Structure and organization of both human *PDGFRA* and *KIT* genes are very similar and could derive from a common ancestral gene. PDGF receptor α is member of the protein tyrosine kinase family subclass III, similar to that of the KIT protein. Chompret et al. described a French family in which 5 individuals had GISTs (age at onset 40–61 years) with germline mutation in *PDGFRA* gene [123]. A 22-year-old patient with multiple GISTs and small intestinal polyps, fibroid tumors, and lipomas was also described in association with V561D germline *PDGFRA* mutation [124]. A unique phenotype including coarse facies and skin, broad hands and feet, and previously undescribed premature tooth loss was described in a family with four first-degree relatives that harbor a *PDGFRA* exon 18 (D846V) germline mutation. The index patient presented with multiple small bowel inflammatory fibroid polyps (IFPs) and has a gastric gastrointestinal stromal tumor (GIST) [125].

### Genes Involved in the Metabolic Mitochondrial Pathway

#### *SDHx* (*SDHA*, *SDHB*, *SDHC*, and *SDHD*) (Succinate DeHydrogenase Complex)

Germline alterations in the *SDHB*, *SDHC*, and *SDHD* genes and, to a lesser extent, the *SDHA* gene predispose to hereditary phaeochromocytoma and/or paraganglioma (PPGL) [126]. Germline mutations in *SDHx* genes are responsible for approximately 20% of cases of PPGL and can also be associated with the presence of other *SDHx*-related tumors including renal cell carcinoma (RCC), GIST, and thyroid and pituitary tumors.

#### *FH* (Fumarate Hydratase)

*FH* inactivating mutations can cause hereditary leiomyomatosis and renal cell cancer (HLRCC), a hereditary cancer syndrome that follows an autosomal dominant inheritance pattern with incomplete penetrance [127]. The age of onset for HLRCC is typically around adolescence to adult with penetrance increasing with age [128]. It is characterized by the development of multiple tumor types including skin leiomyomas, uterine fibroids, and HLRCC kidney tumors with morphological and clinical features similar to those of type 2 papillary renal cell carcinoma (PRCC2) [127]. In addition to the 3 main tumor types, bladder cancer and Leydig cell tumors of the testis have also been reported in HLRCC patients [129, 130].

### Genes Involved in the PIK3, AKT mTOR/AMPK Pathway

#### *TSC1* (Tuberous Sclerosis Complex 1), *TSC2* (Tuberous Sclerosis Complex 2)

TSC is an autosomal dominant disorder caused by the mutation of one of two tumor suppressor genes, *TSC1* or *TSC2*, and characterized by skin manifestations and formation of multiple tumors in different organs, mainly in the central nervous system. The phenotypic expression can vary over the years, with neurological and cutaneous manifestations being more prevalent in childhood, and kidney and pulmonary involvement more characteristic of adulthood. There is a 6–14% incidence of childhood brain tumors in patients with TSC, of which more than 90% are subependymal giant cell astrocytomas [131]. TSC is associated with a cumulative renal cancer incidence of 2.2–4.4%, higher than the estimated incidence in the general population [131]; the average age at diagnosis is 28 years, with occasional early childhood cases [132].

#### *TMEM127* (TransMEMbrane Protein 127)

Loss-of-function alterations in the tumor suppressor *TMEM127* have been detected in familial pheochromocytomas and paragangliomas (PCC/PGL) and associated with increased risk for RCC [133, 134].

#### *STK11* (Serine/Threonine Kinase 11)

Peutz-Jeghers (PJ) syndrome is an autosomal dominant disorder caused by germline mutations of the *STK11* gene and characterized by melanocytic macules of the lips, multiple gastrointestinal hamartomatous polyps, and an increased risk for various neoplasms, including gastrointestinal cancer [135, 136]. Cumulative risk for all cancer was 93% from ages 15 to 64 years old with a significant increase for esophagus, stomach, small intestine, colon, pancreas, lung, breast, uterus, and ovary malignancies [136].

#### *PTEN* (Phosphatase and Tensin Homolog)

Individuals with germline mutations of the *PTEN* tumor suppressor gene have diverse phenotypic features affecting multiple systems, with the primary clinical concern of high lifetime risks of cancer. Elevated risks of breast, thyroid, endometrial, colorectal, and kidney cancers and melanoma were found. The particularly elevated penetrance of breast cancer in females with *PTEN* mutations is noted, beginning around age 30 and rising to an estimated 85% lifetime risk. *PTEN*-related endometrial cancer risk begins at age 25 rising to 30% by age 60, whereas for thyroid cancer, risk begins at birth and continues lifelong. Risks of colorectal and kidney cancers begin around age 40, with a lifetime risk of 9% and 34% respectively. For melanoma, the earliest reported age of onset was 3 years [137].

### Genes Involved in the Wnt/β-Catenin Pathway

#### *APC* (Adenomatous Polyposis Coli)

The *APC* gene encodes a tumor suppressor protein that acts as an antagonist of the Wnt signaling pathway. Defects in this gene cause familial adenomatous polyposis (FAP), an autosomal dominant pre-malignant disease that usually progresses to colorectal cancer. Additionally, in infants and toddlers, there is an increased risk of hepatoblastoma, while in teenagers and adults, duodenal carcinomas, desmoid tumors, thyroid cancer, and medulloblastoma are more common in FAP than in the general population [138, 139].

#### *CDH1* (CaDHerin 1)

*CDH1* is a tumor suppressor gene that is required to maintain cell adhesion, cell polarity, and cell survival signaling. Mutation or transcriptional silencing of the *CDH1 gene* is associated with hereditary diffuse gastric cancer. These patients have a 70% lifetime risk of gastric cancer in males and 56% in females with a median age of 38 years (range 14–69 years). Women additionally have a 42% lifetime risk of lobular breast cancer. The current management for identified carriers includes prophylactic total gastrectomy between the ages of 20 and 40 years, and the initiation of high-risk breast cancer screening with annual mammography and MRI at ages 30–35 years for female carriers [140, 141].

### Genes Involved in the BMP Signaling Pathway

#### *BMPR1A* (Bone Morphogenetic Protein Receptor Type 1A), *SMAD4* (Mothers Against Decapentaplegic Homolog 4)

Germline mutations in *SMAD4* and *BMPR1A* genes have been identified to cause juvenile polyposis syndrome (JPS). It is a rare autosomal dominant hereditary disorder characterized by the development of multiple distinct juvenile polyps in the gastrointestinal tract with an increased risk of colorectal cancer [142]. The reported age at diagnosis of JPS was similar for both *SMAD4* and BMPR1A pathogenic variant carriers (median 28 and 25 years, respectively). The incidence of colorectal cancer is 17–22% by age 35 years and approaches 68% by age 60 years. The median age at diagnosis is 42 years. The incidence of gastric cancer is 21% in those with gastric polyps [143].

### Genes Encoding for Proteins of the SWI/SNF Complex

#### *SMARCB1* (SWI/SNF-Related, Matrix-Associated, Actin-Dependent Regulator of Chromatin, Subfamily b, Member 1), *SMARCA4* (SWI/SNF-Related, Matrix-Associated, Actin-Dependent Regulator of Chromatin, Subfamily A, Member 4)

Carriers of heterozygous constitutional mutations of *SMARCB1* or *SMARCA4* are prone to develop rhabdoid tumors (RT), which have been clinically named as rhabdoid tumor predisposition syndromes 1 (RTS1) and 2 (RTS2), respectively [144, 145]. The manifestation of RTS1 occurs at a very early age, with a median of 5.5 months in children with *SMARCB1* germline mutations, but is very rare in older children or adults [146].

RTPS2 is a complex familial disorder with an autosomal dominant pattern of inheritance with variable penetrance predisposing to formation of tumors that develop in the brain, spine, lung, bladder, pelvis, kidney, or ovary of young children or adults [147]. Genetic profiling has demonstrated these mutations in small cell carcinoma of the ovary and hypercalcemic type (SCCOHT) and *SMARCA4*-deficient undifferentiated uterine sarcoma, as well as atypical teratoid rhabdoid tumors, malignant rhabdoid tumors, and aggressive *SMARCA4*-deficient thoracic sarcomas [148, 149].

### Other Genes

#### *COL7A1* (Collagen Type VII Alpha 1 Chain)

Mutations in *COL7A1* cause the severe inherited blistering disorder recessive dystrophic epidermolysis bullosa (RDEB) affecting skin and mucosae, associated with a greatly increased risk of skin cancer [150]. In the severe generalized subtype of RDEB (Hallopeau-Siemens RDEB), recurrent blistering leads to extensive scarring with a cumulative risk of squamous cell carcinoma (SCC) of 70% by age 45 [151].

#### *DICER1* (Double-Stranded RNA-Specific Endoribonuclease)

The DICER1 syndrome (OMIM 606241) is an autosomal dominant cancer predisposition disorder that is associated with a variety of benign and malignant tumors, including pleuropulmonary blastoma, cystic nephroma, Sertoli-Leydig cell tumors, multinodular goiter, thyroid cancer, rhabdomyosarcoma, and pineoblastoma [152].

#### *EXT2* (Exostosin Glycosyltransferase 2)

Germline mutations in *EXT2* are causative for hereditary multiple exostoses (HME), also called multiple osteochondromas (MO). The main complication in HME is malignant transformation of an osteochondroma (exostosis) into chondrosarcoma, which is estimated to occur in 1–3% of the HME cases [153, 154].

#### *GJB2* (Gap Junction Protein Beta 2)

*GJB2* is mostly known for being associated with syndromic hearing loss, for example, keratitis-ichthyosis-deafness (KID). It has been reported that these KID patients with germline *GJB2* mutation have increased risks of developing epithelial malignancies, for example, 19% occurrence of squamous cell carcinoma of the skin and oral mucosa compared to the normal population [10••].

#### *HABP2* (Hyaluronan Binding Protein 2)

*HABP2* G534E variant functions as a dominant-negative tumor suppressor gene and is a susceptibility gene for familial papillary thyroid cancer [155]. Nevertheless, this association was not confirmed in several subsequent studies [156].

#### *MUTYH* (MutY DNA Glycosylase)

Biallelic pathogenic variants in the *MUTYH* gene cause *MUTYH*-associated polyposis (MAP). MAP is characterized by the presence of 15–100 colorectal polyps and an increased risk of colorectal adenomas and carcinomas [157]. Patients are diagnosed with MAP at a mean age of 45–50 years and median age of CCR onset is 48 [158]. Moreover, carriers of biallelic mutation have an increased risk of ovarian cancer, urinary bladder cancer, cancer of the upper gastrointestinal tracts, breast cancer, endometrial cancer, and skin cancer. Carriers of monoallelic mutation have an approximately 2.5-fold increased risk of CRC compared with the general population [159], but the risk of developing extraintestinal cancer in heterozygotes is still unclear [157].

#### VHL (Von Hippel-Lindau)

Germline inactivation of the VHL tumor suppressor gene causes the von Hippel-Lindau hereditary cancer syndrome (MIM 193300). Common VHL-associated clinical manifestations include central nervous system hemangioblastoma, renal cell carcinoma or renal cyst, retinal angioma, pancreatic tumor or cyst, pheochromocytoma and paragangliomas, endolymphatic sac tumor, and epididymis or broad ligament cystadenoma [160]. Patients may be affected by cancers from childhood and throughout their lifetime [161]. In a recent analysis of a large cohort of Chinese VHL families, the mean ages at onset for central nervous system hemangioblastoma and renal cell carcinoma were 41.1 ± 9.1 (range = 29–62) and 37.4 ± 12.9 (range = 23–65), respectively [160].

## Genetic Counseling and Testing in AYA

Genetic counseling involves three consecutive steps: pre-test counseling, genetic testing, and post-test counseling [42, 162]. Each of these stages should be carried out by a healthcare professional with expertise and experience in cancer risk assessment and management of individuals with an inherited predisposition to cancer [163]. The first step includes an evaluation of patient’s needs and concerns, a detailed collection of personal and family cancer history, a discussion on the possible testing results, subsequent management options, inherited cancer risk to relatives and the privacy of genetic information, and, finally, a written informed consent [164, 165]. The pre-test counseling also guides the clinician towards the most appropriate test to order. The indication to genetic testing is based on the features of an individual’s personal or family medical history. Particularly, age at onset of the tumor, recurrence of specific cancers in the same person or family (e.g., breast, ovarian or colon cancers), unusual cases of cancer (e.g., male breast cancer), the presence of less frequent tumor histotypes (e.g., medullary thyroid carcinoma or triple-negative breast cancer), or birth defects that are known to be associated with inherited cancer syndromes (e.g., neurofibromas). For the most known syndromes, testing criteria are published and universally recognized, such as the Amsterdam criteria for the Lynch syndrome [166] or the Chrompret criteria for Li-Fraumeni syndrome [167]. However, for most of syndromes, testing criteria vary among institutions.

The introduction of multigene testing based on next-generation sequencing (NGS) technology allowed to simultaneously analyze a set of cancer predisposition genes. It is sufficient for cancer risk assessment to evaluate genes of established clinical utility that are suggested by the patient’s personal and/or family history [163]. This approach may be more efficient and cost-effective than the previous tests for single syndrome. Notably, when a pathogenic variant of a predisposition gene is identified on tumor genetic testing, a confirmatory germline testing is recommended. Germline genetic testing should be performed by laboratories equipped to provide analytically and clinically valid results [163]. When results of genetic testing are ready, in a post-test counseling, clinicians should discuss results, related risks, and medical management options in the context of personal and family history.

Patients of reproductive age should also be advised about prenatal diagnosis and assisted reproduction and partners should be tested in case of identification of PV/LPV in genes associated with rare autosomal recessive conditions, such as Fanconi anemia [42, 168]. Moreover, the importance of sharing these results with family members should be discussed so they may benefit from this information [163].

Emotional distress following testing is influenced by factors including disease characteristics (e.g., severity, preventability), amount of uncertainty remaining after testing [169], and ethical and religious beliefs. Often, individuals with hereditary cancer predisposition syndromes worry about passing the condition down to their children and trouble starting a family because of employment and insurance discrimination [170]. Questions about availability of prenatal genetic testing, and occurrence, timing, severity, course, and preventability of cancer may reduce or elevate distress in AYA patients. Young age, perception of high risk, pre-existing psychological distress, a passive way of coping, little social support, and family members with cancer were predictive of psychological problems and/or reduced quality of life [171].

Predictive genetic testing in minors, including adolescents, for conditions for which there are limited preventative or therapeutic measures has traditionally been deferred [42, 172, 173]. Parents or guardians should be informed about the risks and benefits of testing, and in case of testing, their permission should be obtained [174]. Ideally and when appropriate, the assent of the minor should be obtained as well. Then, the patient needs to be informed of the test results at an appropriate age. Finally, parents or guardians should be advised that, under most circumstances, a request by a mature adolescent for test results should be honored [173].

As clinical screening recommendations for many AYA CPS are now available, it is important to consider the family’s lived experiences and their perceived challenges and benefits associated with cancer screening. Exploration of these issues allows the identification of appropriate supports, which may be important to ensure ongoing adherence with surveillance recommendations. The psychosocial impact specifically related to cancer surveillance in adolescents with CPS may be particularly problematic from a psychosocial perspective, given that adolescents are in a key developmental stage of life during which uncertainty about their health is less easily managed [175]. A study looking at the experience of adolescents with hereditary cancer predisposition found that self-concept is influenced, but not defined by tumor risk, and that having this diagnosis allowed for new perspectives on health and illness [176]. The family narrative, or the experiences the family has had with the CPS, has been shown to be an important predictor of one’s personal risk perception [177•]. Therefore, the opportunity to receive accurate, updated medical information at regular intervals is important, particularly as adolescents reach an age when they will assume responsibility for their own health [178].

## Conclusions

The identification of germline pathogenic variants in AYA (summarized in Fig. 1) is especially critical given risk of second primary cancers, need for appropriate long-term surveillance, potential reproductive implications, and cascade testing of at-risk family members, and potentially provides novel biomarkers and therapeutic targets. Among young adults with early-onset phenotypes of malignancies typically presenting at later ages, the increased prevalence of germline PVs supports a role for genetic testing irrespective of tumor type.

## Data Availability

Not applicable
